# *Candida guilliermondii* endocarditis in a patient with prosthetic mitral valve: a case report

**DOI:** 10.22034/cmm.2025.345248.1654

**Published:** 2025-08-17

**Authors:** Saravana Priya J K, Gomathi Manju Balasubramaniam, Ramani C P

**Affiliations:** Institute of Microbiology, Rajiv Gandhi Government General Hospital, Madras Medical College, Chennai, Tamil Nadu, India

**Keywords:** Antifungal agents, *Candida guilliermondii*, Endocarditis, Heart valve prosthesis, Infective endocarditis

## Abstract

**Background and Purpose:**

*Candida* endocarditis, though rare, presents significant risks, especially in prosthetic valve recipients. *Candida guilliermondii* is an uncommon causative pathogen, necessitating a high index of suspicion, advanced diagnostics, and prompt antifungal therapy to improve patient outcomes.

**Case Presentation:**

A 57-year-old male with a history of rheumatic heart disease and prior mitral valve replacement presented with fever, chills, and generalized body aches for 10 days. Laboratory investigations revealed microcytic hypochromic anemia, while echocardiography demonstrated prosthetic valve vegetation with mild to moderate aortic regurgitation. Blood cultures confirmed *C. guilliermondii* infection.

**Conclusion:**

Early recognition, multidisciplinary collaboration, and tailored antifungal therapy are pivotal for favorable outcomes in *C. guilliermondii* prosthetic valve endocarditis. This case highlights the importance of comprehensive diagnostics and vigilant patient management to mitigate complications and improve prognosis.

## Introduction

Infective endocarditis (IE) remains a formidable clinical challenge, characterized by the microbial colonization of the endocardial surface of the heart, frequently resulting in grave morbidity and mortality. While bacterial etiologies predominate, fungal endocarditis–though relatively infrequent–presents significant therapeutic complexities owing to its notoriously high mortality rates and the intricacies of antifungal treatment regimens. Among fungal pathogens, *Candida* species hold particular clinical relevance, with *C. guilliermondii* emerging as a rare but increasingly reported causative agent of IE [ 1 ].

*Candida guilliermondii*, a non-albicans *Candida* species, is predominantly recognized in immunocompromised individuals and those with prosthetic heart valves or intravascular medical devices. Fungal endocarditis, albeit constituting only 1–6% of all IE cases, has dire prognostic implications, with *Candida* species accounting for approximately 2% of these infections. The associated mortality rates remain alarmingly high, ranging from 30% to 80% [ 2 ].

The diagnostic conundrum of fungal IE stems from its nonspecific clinical manifestations, necessitating advanced imaging modalities, such as echocardiography, and rigorous microbiological assessments to ensure precise identification. Current therapeutic paradigms underscore the indispensability of surgical intervention in tandem with prolonged antifungal administration. Echinocandins and amphotericin B have demonstrated superior efficacy in the management of fungal IE, yielding improved clinical outcomes [ 3 ].

This particular case is noteworthy due to the convergence of predisposing cardiac pathology -rheumatic heart disease with mitral stenosis- a prior mitral valve replacement, and the subsequent development of *C. guilliermondii* endocarditis, thereby posing substantial diagnostic and therapeutic hurdles [ 4 ].

## Case Presentation

A 57-year-old male, diagnosed 15 years prior with rheumatic heart disease and mitral stenosis, underwent mitral valve replacement a year ago. The patient presented with a 10-day history of persistent fever, intermittent chills, and generalized body aches. Informed verbal consent was obtained from him before disclosing patient details.

Upon examination, the patient exhibited signs of systemic illness. Cardiovascular auscultation revealed an ejection click, indicative of valvular pathology. Hematological analysis demonstrated microcytic hypochromic anemia, reinforcing underlying systemic inflammation.

A normally functioning prosthetic valve in the mitral position was observed. Myocardial velocity gradients measured 19 mm Hg (peak) and 15 mm Hg (mean). A highly mobile vegetation with a size of 7 mm was detected. Mild to moderate aortic regurgitation was present, with no tricuspid regurgitation or evidence of abnormal pressure half-time. Left ventricular systolic function was preserved.

**Figure 1:**
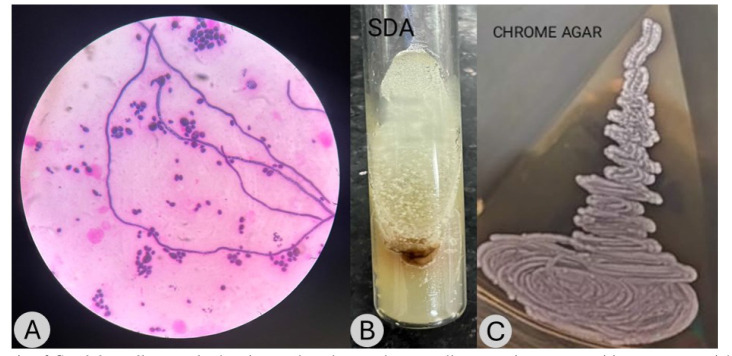
(A) Gram stain of *Candida guilliermondii* showing oval to elongated yeast cells, appearing gram-positive to gram-variable. Pseudohyphae were present but typically sparsely branched. (B) Sabouraud dextrose agar culture of *Candida* guilliermondii showing flat, moist, smooth colonies with a cream to yellow coloration. (C) CHROMagar *Candida* culture of *Candida guilliermondii* showing pink to beige colonies, distinguishing it from other *Candida* species

Further infectious disease workup ruled out bacterial etiologies, with negative Widal and dengue tests. The blood specimen was processed using the BACTEC and VITEK 2 Compact automated system, a widely recognized tool for microbial identification. Following incubation and analysis, the system confirmed the presence of *C. guilliermondii*, enabling precise species-level identification and guiding targeted antifungal therapy. Gram staining of *C. guilliermondii* revealed oval to elongated yeast-like cells (Figure 1A). Pseudohyphae were observed, though they exhibited limited and sparse branching. On Sabouraud dextrose agar, the isolate formed flat, moist, and smooth colonies with a cream to pale yellow pigmentation (Figure 1B). When cultured on CHROMagar *Candida*, *C. guilliermondii* developed distinctive pink to beige colonies (Figure 1C). Antifungal susceptibility testing performed by the system provided crucial insights into the sensitivity profile of the pathogen. The minimum inhibitory concentrations for voriconazole, caspofungin, fluconazole, micafungin, amphotericin B, and flucytosine were < 0.12 µg/mL, 0.5 µg/mL, 1 µg/mL, 0.5 µg/mL, 0.5 µg/mL, and < 1 µg/mL, respectively. All tested antifungal agents showed sensitivity, guiding the selection of an appropriate therapeutic regimen. Based on these results, a tailored antifungal treatment strategy was initiated, ensuring optimal management of *C. guilliermondii* endocarditis and improving patient prognosis. 

The patient underwent a two-week regimen of combination antifungal therapy, incorporating caspofungin and fluconazole, to target the infection effectively. Caspofungin was administered at 70 mg on the first day, followed by 50 mg daily, while fluconazole was administered at 400 mg (6 mg/kg) daily. A repeat blood culture subsequently returned negative, confirming microbial clearance. Given the high recurrence risk associated with prosthetic valve infections, fluconazole (400 mg/day) was extended for an additional six weeks as a suppressive regimen to prevent relapse and ensure sustained eradication of the pathogen. Surgical intervention was not performed in this case. While fungal endocarditis, especially on prosthetic valves, often requires surgery, the non-surgical approach was selected due to clinical stability, response to antifungal therapy, and comorbidities. The patient showed a favorable response to caspofungin and fluconazole therapy without signs of worsening infection. 

Follow-up echocardiography, which was performed for six weeks post-treatment, showed no residual vegetations, confirming infection resolution. The patient remained clinically stable, with no signs of recurrent fungemia or valvular dysfunction. Long-term monitoring continued with fluconazole suppression therapy to prevent relapse. Timeline of symptoms is demonstrated in Table 1.

**Table 1:** Timeline of symptoms

Date (approximate)	Event
15 years ago	Diagnosis of rheumatic heart disease with mitral stenosis
1 year ago	Performance of mitral valve replacement surgery
10 days ago	Onset of fever and intermittent chills, Development of intermittent body aches
Day 1	Blood cultures drawn, revealing *Candida* species in Gram stain, Performance of echocardiography, showing highly mobile vegetation (7 mm) on the prosthetic mitral valve
Day 3	VITEK identification confirmed *Candida* species; initiation of antifungal susceptibility testing
Day 4 to Day 46	Start of caspofungin and fluconazole therapy. Continued for 6 weeks
Follow-up (6 weeks post-treatment)	Repeat echocardiography showing no residual vegetations, confirming infection resolution; continuation of fluconazole suppression therapy

## Discussion

Fungal IE, particularly *Candida* species-associated infections, remains a formidable clinical challenge due to its high morbidity and mortality rates. While *Candida albicans* is the most frequently implicated species, *C. guilliermondii* has been increasingly documented in prosthetic valve recipients and immunocompromised individuals. Rarity of *C. guilliermondii* in endocarditis necessitates heightened diagnostic awareness and a tailored therapeutic approach.

Clinical literature underscores the necessity of a multimodal treatment strategy, integrating surgical valve replacement with prolonged antifungal therapy. Echinocandins, such as caspofungin, and polyenes, notably amphotericin B, have demonstrated superior efficacy in eradicating fungal endocarditis. However, fluconazole remains a viable option for susceptible strains, particularly in cases where long-term suppressive therapy is required [ 5 ].

Clinical presentations of the patient, diagnostic findings, and microbiological confirmation of *C. guilliermondii* emphasized the urgency of an expedited and methodical treatment approach. Successful resolution of infections following targeted antifungal therapy highlights the critical need for early fungal pathogen consideration in prosthetic valve recipients presenting with infective endocarditis [ 6 ].

Given the diagnostic challenges, standard blood cultures may be insufficient, necessitating advanced methods, such as VITEK, Matrix-Assisted Laser Desorption/Ionization Time-of-Flight, and polymerase chain reaction for precise identification. Prior antimicrobial exposure further complicates fungal detection, while biomarkers, such as β-D-glucan, enhance diagnostic accuracy [ 7 ].

A combination of caspofungin and fluconazole is effective in infective endocarditis caused by *Candida* species, as it targets both cell wall and membrane integrity. Studies suggest that combination therapy improves fungal clearance and reduces resistance risk [ 8 , 9 ].

Recent case reports reinforce the importance of early recognition and aggressive management. A documented case of *C. guilliermondii* native left-sided valve endocarditis demonstrated the efficacy of surgical intervention combined with fluconazole therapy, leading to sustained patient recovery. Similarly, reports of prosthetic valve *C. parapsilosis* endocarditis highlight the necessity of both antifungal treatment and timely surgical intervention.

One of the strengths of this study was a meticulous diagnostic approach integrating echocardiography and blood cultures that facilitated timely identification of the causative pathogen. Prompt initiation of targeted antifungal therapy proved instrumental in mitigating disease progression. A collaborative effort among cardiologists, infectious disease specialists, and laboratory experts ensured holistic patient management.

Among the limitations of this study was the initially nonspecific symptomatology that rendered early clinical suspicion challenging. Moreover, the rarity of *C. guilliermondii* infections may have contributed to diagnostic delays. Limited access to advanced molecular diagnostics may constrain timely pathogen identification in certain clinical settings.

## Conclusion

This case underscored the complexity of fungal infective endocarditis, particularly with rare pathogens, like *C. guilliermondii*, in prosthetic valve recipients. Early detection, advanced diagnostics, and a targeted antifungal regimen were crucial in achieving a favorable outcome. Given the high mortality rates, a vigilant, multidisciplinary approach is essential for optimizing patient recovery and ensuring effective management of such infections.
